# Early brain cognitive development in late preterm infants: an event-related potential and resting EEG study

**DOI:** 10.1186/s13052-023-01567-9

**Published:** 2024-02-14

**Authors:** Qinfen Zhang, Xuan Dong, Wenjie Jin, Jiaojiao Fan

**Affiliations:** Children’ s Health Research Center, Changzhou Children ’ s Hospital of Nantong University, 468 Yanling Middle Road, Tianning District, Changzhou, 213003 Jiangsu China

**Keywords:** Late preterm infants, Event-related potential, Resting EEG, Brain cognitive function, Prediction

## Abstract

**Background:**

Late preterm infants (LPIs) are at risk of neurodevelopmental delay. Research on their cognitive development is helpful for early intervention and follow-up.

**Methods:**

Event-related potential (ERP) and resting electroencephalography (RS-EEG) were used to study the brain cognitive function of LPIs in the early stage of life. The Gesell Developmental Scale (GDS) was used to track the neurodevelopmental status at the age of 1 year after correction, and to explore the neurophysiological indicators that could predict the outcome of cognitive development in the early stage.

**Results:**

The results showed that mismatch response (MMR) amplitude, RS-EEG power spectrum and functional connectivity all suggested that LPIs were lagging behind. At the age of 1 year after correction, high-risk LPIs showed no significant delay in gross motor function, but lagged behind in fine motor function, language, personal social interaction and adaptability. The ROC curve was used to evaluate the predictive role of MMR amplitude in the brain cognitive development prognosis at 1 year, showing a sensitivity of 80.00% and a specificity of 90.57%. The area under the curve (AUC) was 0.788, with a P-value of 0.007.

**Conclusions:**

Based on our findings we supposed that the cognitive function of LPI lags behind that of full-term infants in early life. Preterm birth and perinatal diseases or high risk factors affected brain cognitive function in LPIs. MMR amplitude can be used as an early predictor of brain cognitive development in LPIs.

**Trial registration:**

This clinical trial is registered with the Chinese Clinical Trial Registry (ChiCTR). Trial registration number: ChiCTR2100041929. Date of registration: 2021-01-10. URL of the trial registry record: https://www.chictr.org.cn/.

## Background

Late preterm infants (LPIs) refer to preterm infants born between 34 and 37 weeks of gestational age, accounting for approximately 70% of preterm infants. The incidence of neurodevelopmental delay or defects in LPIs is 36% higher than that in full-term infants [1], and LPIs have a high risk of disability, academic failure, behavioral problems, language delay, social communication and cognitive deficits, and even death [2]. Cheong used Bayley Scales of Infant Development (BSID) to conduct developmental follow-up experiments in 198 LPIs and 183 full-term infants (followed up to the corrected age of 2 years), and found that compared with full-term infants, LPIs showed poor performance in cognitive, language, motor and social-emotional abilities [3]. Woythaler found that LPIs were significantly behind full-term infants in terms of expression, reading and calculation [4]. Therefore, LPIs have various neurodevelopmental risks, especially developmental delay in various high-level cognitive abilities. Cognitive abilities develop rapidly in the first years of life, with cognitive flexibility, language and behavioral control, as well as social adaptation and emotional adjustment being particularly important in learning social survival [5]. Therefore, active attention should be paid to this group, and early assessment and intervention should be carried out for serial follow-up.

Sutton proposed the concept of event-related potential (ERP) through the classic Oddball experimental paradigm in the 1960s. ERP is considered a “window” of mental activity, also known as cognitive potential, as it can quantitatively analyze the neuropsychological response of cognitive tasks. Potentials evoked by auditory stimuli are called auditory event-related potentials (aERP), which can provide insights into specific aspects of cognition, memory, and decision-making. Existing studies have pointed out that auditory cognitive memory is a brain function that appears early in human development [6]. Fetal hearing develops in the third trimester of gestation, and human auditory nerve conduction begins to develop in the fetal period and becomes mature after birth [7]. Therefore, an aERP study was conducted on preterm infants with auditory stimulation to determine the attention processing and discrimination ability of the brain to sound stimulation, in order to evaluate the development of brain cognition.

Mismatch negativity (MMN) is a special endogenous component of aERP discovered by Naatanen [8]. MMN is the negative wave distributed in the forehead and center of the brain. It is generally produced 100 to 250 ms after differential stimulation. The MMN of infants is very different. In addition to generating the same negative wave as that of adults, it can also generate positive waves. Therefore, some studies suggest that the MMN of infants should be called mismatch response (MMR) [9, 10]. MMR studies in infants have shown that positive waves are an immature feature, and associations have been found between a variety of lesions and pathological MMR (MMN). Lovio [11] and Huttunen-Scot [12] recorded MMR abnormalities in children at risk of dyslexia and attention deficit disorder, respectively. Tanaka reported that neonates with a gestational age of 35–48 weeks could induce MMN similar to that of adults, while preterm infants with a gestational age of 36–37 weeks and neonates with a greater gestational age had significantly prolonged latency and were not sensitive to bias stimuli, which also suggested that the brain cognitive function of LPIs was in an immature stage [13]. Bisiachi conducted a MMR study on preterm infants aged 23–29 weeks and 30–34 weeks at 35 weeks of corrected gestational age. Both groups of preterm infants produced significant negative waves, but the amplitude of MMR in the younger gestational age group was significantly lower than that in the older gestational age group, suggesting that the amplitude of MMR was positively correlated with gestational age, that is, the greater the gestational age, the more mature the amplitude, reflecting the more mature cerebral cortex [14].

Studies have also assessed brain cognitive function in preterm infants through verbal stimulation tasks. Rago used the Oddball paradigm to analyze the speech change discrimination and stress change processing ability of preterm infants and normal full-term infants at almost 36 weeks. The results showed that although preterm infants possess the capability for speech discrimination [15], they exhibit a developmental delay in processing the intricacies of speech compared to their term counterparts. These studies indicate that MMR serves as an efficacious instrument for evaluating early-life cognitive functions specifically in LPIs [16, 17], and may act as a viable biomarker for assessing cognitive developmental trajectories in this population.

Electroencephalography (EEG) is a continuous measurement of cortical rhythm, which can be used as an important data source for quantitative measurement of cortical dynamics [18]. Due to the advantages of high time resolution, non-invasiveness, low cost and low requirements of the acquisition environment, it has become an important indicator of brain cognitive development [19], which can monitor and predict the early injury factors of brain function and the long-term prognosis of the nervous system [20]. Resting state EEG (RS-EEG) records ongoing EEG signals without performing specified cognitive tasks. It is the most basic and essential state among various complex brain states, which can reflect the inherent activity pattern of the brain and is the core of various cognitive activities [21]. RS-EEG is very suitable for the study of cognition throughout the life cycle and individual studies of people with atypical development [22]. Brito found that there was a significant correlation between resting EEG power and 15-month cognitive ability, especially declarative memory and auditory comprehension [23]. Williams reported a significant correlation between RS-EEG and BSDI-III at 18 months in 13 full-term neonates with congenital heart disease [24]. Benasich studied the RS-EEG of children aged 16, 24 and 36 months and showed that preschool gamma power was significantly correlated with language and cognition [25]. These studies prospectively examined the association between RS-EEG and cognitive development. In this study, we combined these two neuroelectrophysiological tools to explore the cognitive function of LPI brain.

In pediatric neurodevelopmental research, the quest for early biomarkers indicative of atypical cognitive development in high-risk infants, particularly those born preterm, remains a key focus. While traditional approaches have leaned heavily on clinical and behavioral indicators [26], the innovative potential of tools like Event-related Potentials (ERP) and resting-state EEG is only starting to be explored [27]. Although comprehensive studies leveraging these neurophysiological measures remain relatively scant, pioneering works have begun to underline their potential role in the early identification of atypical cognitive developmental trajectories in vulnerable infant populations [28].

## Methods

### Participants

LPIs hospitalized in the Department of Neonatology, Changzhou Children’s Hospital of Nantong University from September 2019 to December 2021 were randomly selected as the research subjects, and full-term infants without brain injury were included as the control group. All subjects passed hearing screening of both ears. The LPIs included in the study were gestational age infants born between 34 and 36^+ 6^ weeks. According to the presence or absence of perinatal diseases or high-risk factors, LPIs were divided into high-risk and low-risk LPIs. High-risk LPIs were associated with perinatal diseases (such as hypoglycemia, severe metabolic acidosis, frequent apnea, sepsis, intracranial infection, severe hyperbilirubinemia, etc.) that may lead to brain damage. Low-risk LPIs were defined as those without perinatal disease. The control group consisted of infants with mild respiratory or gastrointestinal infections at the same time, without brain injury. In the control group, the gestational age was 39–41^+ 6^ weeks, birth weight was between 2500 and 4000 g, and the Apgar score was more than 7 points at 1 and 5 min.

The exclusion criteria included craniofacial malformations, congenital brain dysplasia, moderate to severe hypoxic ischemic encephalopathy (HIE), genetic metabolic diseases, etc. Excessive amplitude of head movement, environmental factors and other causes of excessive EEG artifacts were excluded.

### Study design

This study was registered at the Chinese Clinical Trial Center (ChiCTR2100041929).

RS-EEG and ERP were recorded when the LPIs were stable. After feeding, the newborn was placed in a comfortable position on a cot or held, in order that he/she was in a quiet and awake state. No sedatives were administered to ensure the weak light and sound insulation of the experimental environment, and the room temperature was controlled at 24–26℃.

A Neuracle portable wireless digital EEG system (NSW332) was used, and recording electrodes were placed according to the international 10–20 system, and EEG signals were recorded with a 19 electrode cap.

The Oddball paradigm was selected for the experiment, with stimulus pairs of 2000 Hz short pure tone and 1000 Hz short pure tone. The experiment was divided into two blocks, each with 500 trials, in which the proportion of deviant stimulation was 20% and the standard stimulation was 80%, both lasted for 100 ms, and the two blocks were balanced. The interval between stimuli was randomly 600–800 ms. Sound was below 60 dB Sound Pressure Level (SPL), and stimulation was controlled using E-Prime software. A sound box was placed 20 cm away from both ears of the subjects to play the sound.

Evaluation of cognitive function inLPIsat 1 yearwas performed using the Gesell Developmental Scale (GDS). The GDS is utilized as part of a broader arsenal of neurodevelopmental assessment tools, each contributing unique insights into the multifaceted landscape of early brain function development. The GDS includes five subscales: gross motor function, fine motor function, adaptability, language, and social skill evaluation. The developmental quotient (DQ) was calculated as follows: (development age/actual age)×100. DQ > 130 is excellent, 115 ≤ DQ ≤ 129 is smart, 85 ≤ DQ ≤ 114 indicates a normal level, 76 ≤ DQ ≤ 85 indicates edge state, 55 ≤ DQ ≤ 75 indicates mild developmental delay, 40 ≤ DQ ≤ 54 indicates moderate delay, 25 ≤ DQ ≤ 39 indicates severe delay, and DQ < 25 indicates profound neurological delay.

### Data processing

CPz is an online reference electrode with a sampling rate of 1000 Hz. The impedance between the electrode and the scalp was less than 5 kΩ, and the filtering bandpass for online recording was 0.01 to 70 Hz. MATLAB R2019b software was used for off-line analysis. The bandpass (0.01-45 Hz) was filtered, and the EEG artifacts were removed, and the whole brain average was selected as the re-reference, and 200 ms before stimulation was used as the baseline correction. The ERPLAB toolbox was used to extract the ERP components by the average superposition technique, and MMR was obtained by subtracting deviation stimulus and standard stimulus ERP waves. After pretreatment, the average effective trials ratio for each task was 83%. Based on the observation of the current evoked brainwave shape and previous related studies, Cz channel analysis was selected to compare the average amplitude of MMN components, and the analysis time window selected was 200–300 ms.

The spectrum of the preprocessed EEG data was calculated using the Pwelch function of MATLAB R2019b. 120 segments were selected, the window function was set as 4 S, no overlap, NFFT = 2000, and the power spectrum of each channel was extracted. The results were then converted by 10*log20. The frequency range of analysis was 0.01-8 Hz: δ(0.01-4 Hz),θ(4–8 Hz). The spectral coherence (COH) of RS-EEG was calculated, and the coherence matrix of 1–8 Hz was calculated using toolkit HERMES and GRETNA. False Discovery rate (FDR) correction and network-based statistic (NBS) correction were performed.

### Statistical analysis

SPSS 23.0 was used for statistical analysis of the experimental data. MMR amplitude and RS-EEG power spectrum among the three groups were statistically analyzed by one-way analysis of variance (ANOVA), and the Bonferroni method was used for multiple comparisons. MATLAB 2019b was used for the FDR calibration test and NBS calibration test. A receiver operating characteristic (ROC) curve was drawn, and the sensitivity, specificity and area under the ROC curve were calculated. *P* < 0.05 was considered statistically significant.

## Results

### Baseline characteristics of the study participants

A total of 75 neonates were included in this experiment, of which 6 were terminated due to crying and agitation and the data were incomplete, and 4 were excluded due to large artifacts. The general information of each group is shown in Table 1. There were no significant differences in gender and Apgar score at 5 min among the three groups (*P* > 0.05). There were significant differences in gestational age, birth weight and head circumference. In order to ensure safety, data were collected when the condition of the subjects was stable. Therefore, there were differences in the age at which the test was carried out among the three groups (*P* < 0.05), but the test was completed within 4 weeks of birth.

**Table 1:** Baseline characteristics of included subjects

Characteristics	high-risk LPIs(n = 19)	low-risk LPIs(n = 20)	Full-term infants(n = 26)	χ^2^/*F*	*P*
Sex-Male n(%)	13(68.42)	14(70.00)	13(50.00)	1.333	0.275
Gestational age(w)	35.10 ± 0.90	35.80 ± 1.10	39.80 ± 0.70	181.640	0.001
Birth weight(g)	2238.51 ± 472.20	2529.51 ± 358.81	3364.21 ± 379.10	49.011	0.001
head circumference(cm)	32.20 ± 3.02	32.62 ± 1.41	34.35 ± 0.91	8.919	0.001
Apgar(5 min)	8.81 ± 0.51	8.94 ± 0.32	9.32 ± 0.18	2.045	0.138
Test age(d)	18.82 ± 8.21	10.92 ± 9.93	12.24 ± 8.15	4.787	0.012

### MMR results

**Fig. 1:**
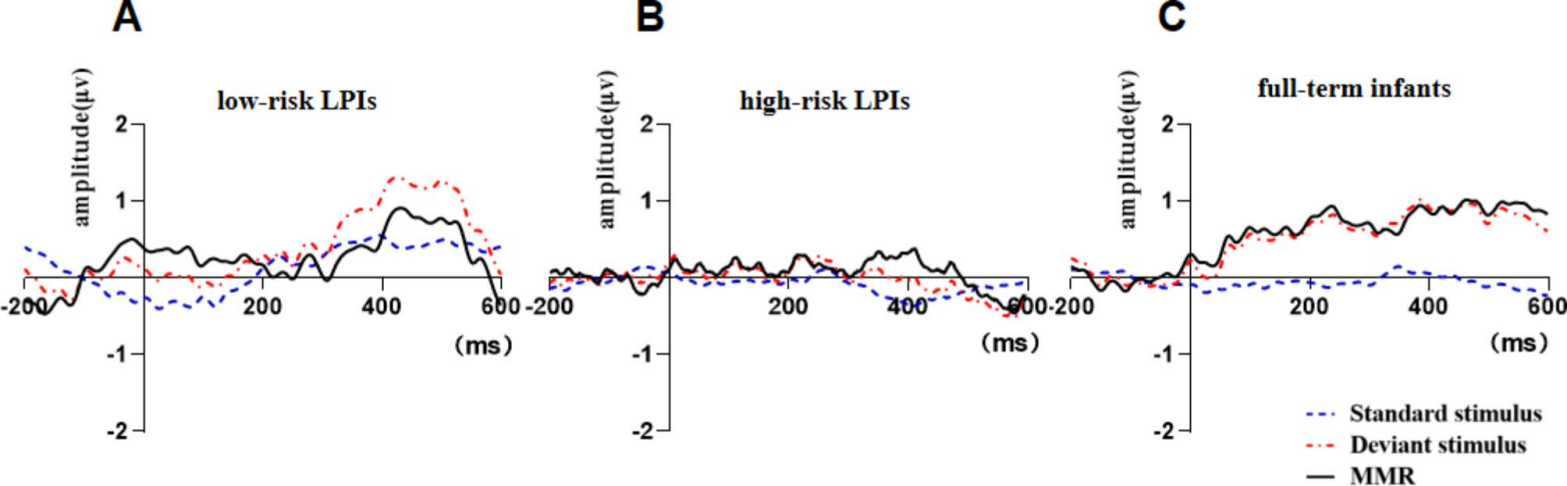
ERP wave of the three groups of subjects in Cz lead. **A** is the low-risk LPIs group, **B** is the high-risk LPIs group, and **C** is the full-term infant group. The blue dashed line is standard stimulus wave, the red dashed line is deviant stimulus wave, and the black solid line is MMR wave

The neural origin of MMR induced by the auditory Oddball paradigm is mostly in the central frontal region, and the Fz lead was selected for this investigation. Figure 1 shows the standard stimulus wave, deviation stimulus wave and their difference waves in the three groups. From the ERP waveform results of the three groups, it can be seen that the full-term infant group had a larger response to deviation stimulus and the amplitude was largest. In addition, the amplitude of deviant stimulation in high-risk LPIs was smallest, and the response was similar to that of standard stimulation, suggesting that the LPI group was less sensitive to deviant stimulation than full-term infants. Figure 1 also shows that both LPIs and full-term infants generate MMR waves under the auditory Oddball paradigm.

According to the results of previous studies on MMR components and ERP waves, 200–300 ms was selected as the time window to analyze the average amplitude within this time window. The amplitudes of low-risk LPIs, high-risk LPIs and full-term infants were 0.09 ± 1.15 µV, 0.13 ± 1.31 µV and 0.98 ± 0.82 µV, respectively. ANOVA showed that the average amplitude of MMR was significantly different among the three groups (*F* = 5.077, *P* = 0.009). Multiple comparisons using the Bonferroni method showed that the average amplitude of MMR in the high-risk LPIs group was significantly smaller than that in the full-term group (*P* < 0.05), and was significantly smaller in the low-risk LPIs group than in the full-term group (*P* < 0.05). However, there was no significant difference in LPIs between the two groups (*P* > 0.05), indicating that the average amplitude of MMR was significantly reduced in both high-risk LPIs and low-risk LPIs.

### RS-EEG results

#### Power spectrum analysis

Table 2 shows that there were statistical differences in the power spectra of δ (0.01-4 Hz) and θ (4–8 Hz) bands among the three groups (*F* = 6.661, *P* = 0.002; *F* = 5.875, *P* = 0.005). Bonferroni multiple comparisons showed that both the δ band and θ band suggested that the power spectrum in the low-risk LPIs group was significantly larger than that in the full-term group and high-risk LPIs group (*P* < 0.05), while the high-risk LPIs group and full-term group showed no statistical differences (*P* > 0.05).

**Table 2:** Comparison of power spectra(20*log10, $\bar x$±s)

High-risk LPIs (n = 19)	low-risk LPIs (n = 20)	Full-term infants (n = 26)	*F*	*P*
14.27 ± 2.56	17.90 ± 43.21	13.46 ± 5.67	6.661	0.002^a^
-7.69 ± 2.73	-3.87 ± 5.37	-6.55 ± 2.21	5.875	0.005^b^

There were significant differences in the power spectra of δ and θ bands among the three groups (*P* < 0.05). a: Multiple comparisons showed that there was a statistical difference between low-risk LPIs group and full-term group (*P* = 0.002), a statistical difference between high-risk LPIs and low-risk LPIs (*P* = 0.029), and no statistical difference between high-risk LPIs and full-term group (*P* > 0.05). b: Multiple comparisons showed that there was a statistical difference between low-risk LPIs group and full-term group (*P* = 0.044), a statistical difference between high-risk LPIs and low-risk LPIs (*P* = 0.005), and no statistical difference between high-risk LPIs group and full-term group (*P* > 0.05)

#### Functional connection analysis

In this study, functional connections between brain regions in the range of 1–8 Hz were constructed. The COH algorithm was selected to obtain the coherence matrix representing the connection coefficient between 19 × 19 leads, and each element in the matrix represented the connectivity size between two leads.

**Fig. 2:**
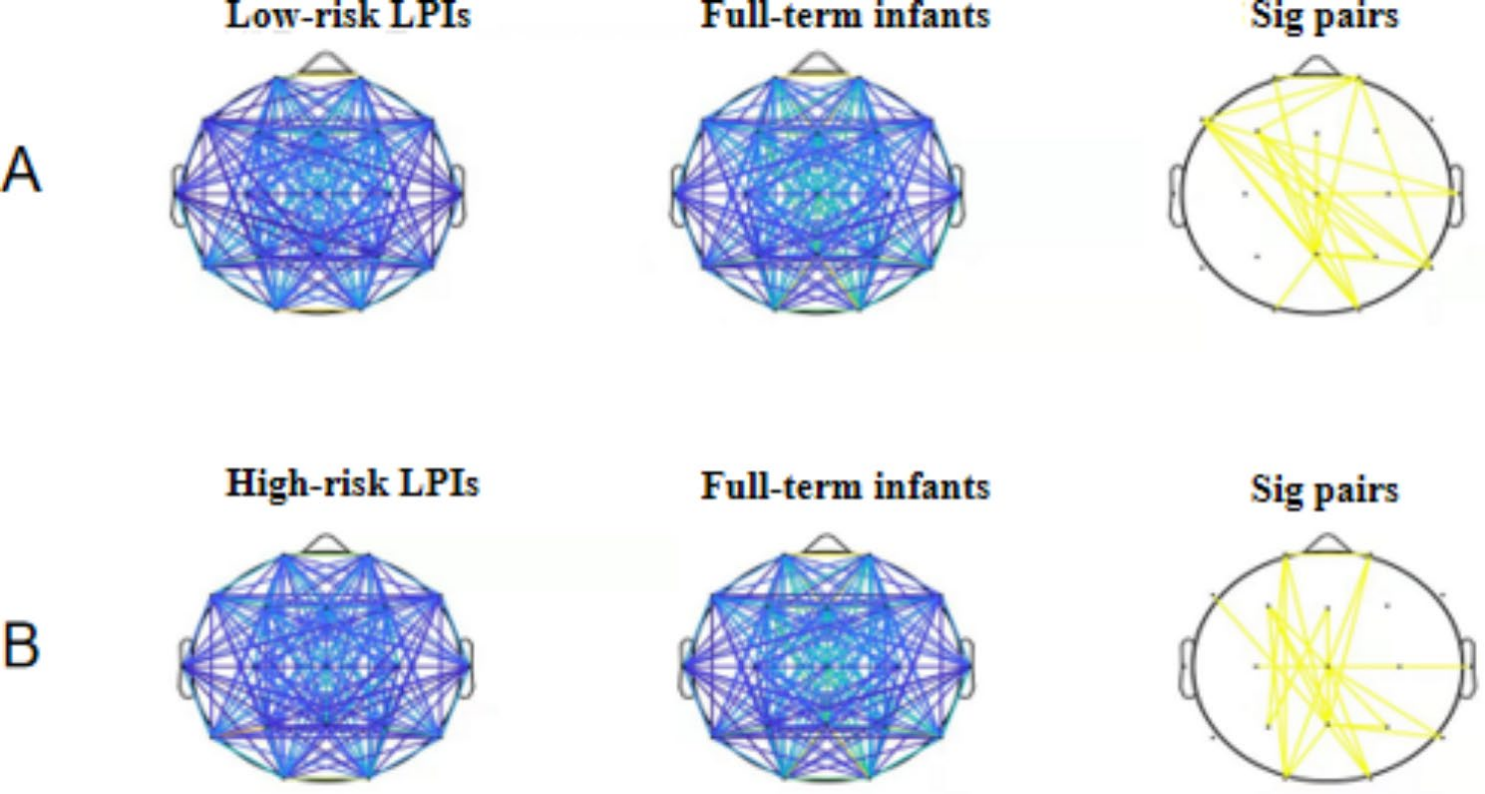
COH and NBS test results. There was no significant difference in connectivity between the low-risk LPIs group and the high-risk LPIs group, which is not shown in the figure above. Sig Pairs indicates connections with significant differences (*P* < 0.05)

The NBS test was performed on the COH results by the NBS toolbox. The results showed that the connectivity of the low-risk LPIs and high-risk LPIs groups was lower than that of full term infants, and functional connectivity of the low-risk LPIs group in the prefrontal parietal region, temporal-central region, left fronto-central region, and parieto-occipital region was significantly decreased (*P* < 0.05). In the high-risk LPIs group, there was a significant decrease in fronto-central and parieto-occipital connectivity (*P* < 0.05) (Fig. 2).

### GDS results

A total of 53 infants in the three groups were evaluated by the GDS up to 1 year after correction, including 17 infants in the high-risk LPIs group, 16 infants in the low-risk LPIs group and 20 in the full-term infant group. There were statistically significant differences between the three groups in fine motor function, language, social skill and adaptability (*P* < 0.05). However, no significant difference in gross motor function was observed (*P* > 0.05). DQ was calculated, and the differences between the three groups were statistically significant (*P* < 0.05), and Bonferroni multiple comparisons showed that DQ was significantly smaller in the high-risk LPIs group than in the low-risk LPIs and full-term groups (*P* < 0.001), but there was no significant difference between the low-risk LPIs group and the full-term group (*P* > 0.05). These results are shown in Table 3.

**Table 3:** Comparison of GDS($\bar x$±s)

group	High-risk LPIs(n = 17)	low-risk LPIs (n = 16)	Full-term infants (n = 20)	*F*	*P*
gross motor	87.16 ± 7.03	88.80 ± 6.64	91.46 ± 4.55	2.945	0.060
fine motor	85.26 ± 5.23	90.25 ± 6.44	94.50 ± 4.06	17.192	<0.001
language	86.74 ± 7.71	89.60 ± 8.24	92.96 ± 4.04	4.845	0.011
social skill	80.11 ± 7.86	91.40 ± 6.24	94.69 ± 4.43	32.451	<0.001
adaptability	82.79 ± 5.59	88.95 ± 6.92	89.12 ± 5.61	7.222	0.002
DQ	84.41 ± 5.38	89.80 ± 5.77	92.55 ± 1.88	18.299	<0.001

### Predictive effect of MMR amplitude on cognitive development of LPIs brain

Drawing from the results of the GDS at 1 year, this study standardizes the prognosis of cognitive development in LPIs. DQ ≤ 75 was established as the threshold for atypical cognitive development, with 5 infants (4 high-risk LPIs and 1 low-risk LPI) falling below this criterion. Furthermore, from 53 participants, abnormal MMR amplitude (-0.59 ± 1.13 uV) was observed in 9 infants, inclusive of 4 from the high-risk LPIs, 4 from the low-risk LPIs, and 1 full-term infant. The remaining cohort exhibited a normal MMR amplitude, averaging 0.67 ± 1.05 uV.

**Fig. 3:**
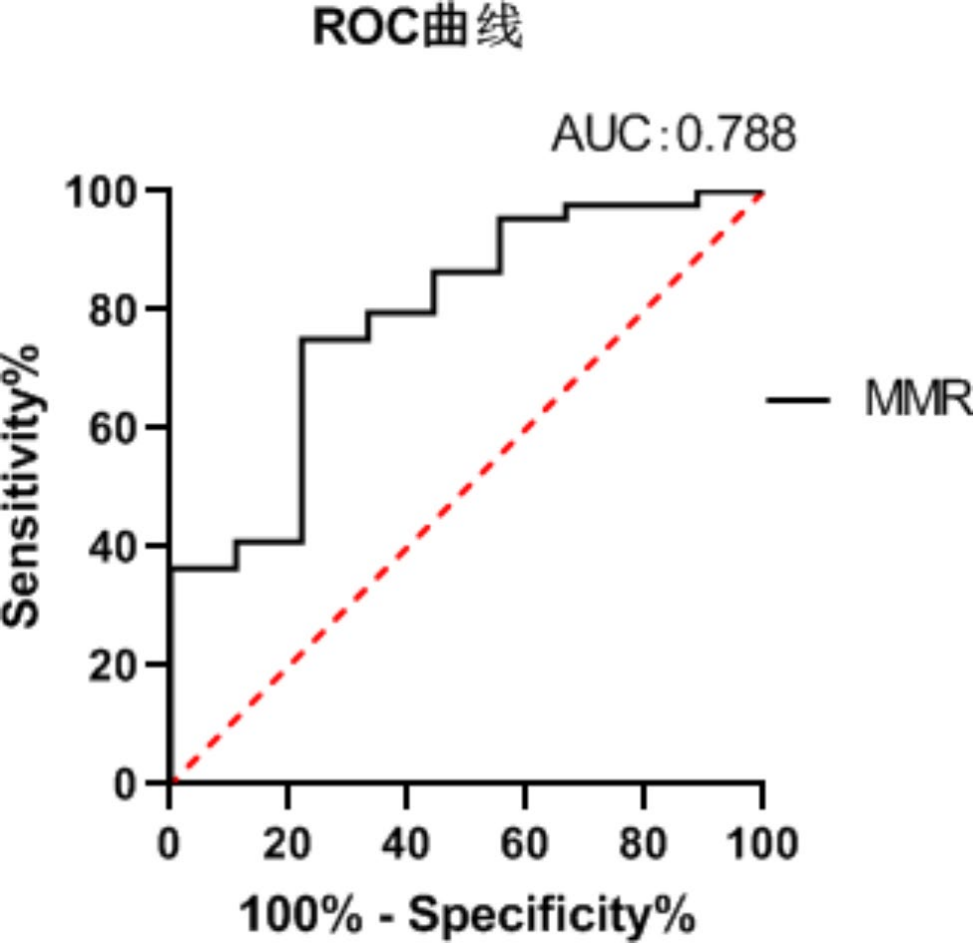
ROC Curve for Predicting Brain Cognitive Development in LPIs Using MMR Amplitude

The ROC curve was used to evaluate the MMR amplitude in predicting prognosis, with a sensitivity of 80.00%, a specificity of 90.57%, and area under curve (AUC) of 0.788, *P* = 0.007 (Fig. 3), suggesting that MMR amplitude could be used as an effective indicator to predict the prognosis of brain cognitive development at the age of 1 year.

## Discussion

The findings of this study demonstrate that LPIs exhibit disparities in various neurodevelopmental indices, as evidenced by diminished MMR amplitude, altered RS-EEG power spectra, and compromised functional connectivity. At one year of corrected age, high-risk LPIs displayed no considerable delays in gross motor abilities; however, they exhibited significant deficits in fine motor skills, linguistic development, interpersonal social interactions, and adaptive capacities.

Cognitive processing ability of auditory tasks was found in both LPIs and term infants, and MMR waves were generated in both LPI groups, which were significantly lower than those in term infants. These results are consistent with those from studies on cognitive brain function in preterm and term infants. Bisiacchi found that cognitive processes were related to gestational age through ERP of very low gestational age (23–29 weeks, n = 20) and low gestational age (30–34 weeks, n = 19) preterm infants, that is, the MMR amplitude of preterm infants with gestational age less than 30 weeks was smaller than that of those with gestational age more than 30 weeks [29]. They proposed the importance of in utero development for the development of cerebral cortical pathways and, as a result, sufficient influence on cognitive function. Diseases in the neonatal period also cause delayed maturation of brain development, thus affecting brain cognitive function. Leipala found that the amplitude of MMR in healthy term infants was significantly higher than that in term infants with brain injury, and the early brain function of term infants with perinatal brain injury was lower than that of healthy term infants [30]. Some studies have suggested that the forward wave is an expression of immaturity [31]. Our study found that the MMR produced by the three groups of infants showed positive waves, so mismatched response ability at the early stage of birth was not mature in term or preterm infants.

Anderson [32] and Noreika [33] proposed that EEG power can be used to measure the cortical rhythm of rapid development and change in brain activity in early life. In our study, RS-EEG power spectrum showed that the power of low-risk LPIs in the δ and θ bands was significantly higher than that of full-term infants, which is consistent with many studies suggesting that the absolute power of the δ wave was significantly negatively correlated with gestational age. Okumura believed that the δ wave spectrum decreased with increased gestational age [34]. Bellanalyzed the power spectrum of 20 healthy preterm infants from 26 to 32 weeks, and found that the spectral power of δ1 (0.5-1 Hz) and δ2 (1–4 Hz) in the frontal lobe (F3-C3 and F4-C4) and parietal lobe (C3-P3 and C4-P4) decreased with increased gestational age, and the absolute power of the δ wave was significantly negatively correlated with gestational age [35]. Tsuhida considered that δ energy plays a dominant role in neonatal cerebral cortex activity, and objective assessment of δ activity will help predict neural outcomes in preterm infants [36]. In addition, the study of θ power is necessary for the cognitive activity of infants. Begus suggested that the θ rhythm in infancy is an indicator of active learning and memory [37]. The power of high-risk LPIs in our study was significantly lower than that in full-term infants in the δ and θ bands. The spectrum characteristics of low-risk LPIs were consistent with gestational age, that is, the power in the δ and θ bands was negatively correlated with gestational age [38], and the spectrum of low-risk LPIs was significantly higher than that of the full-term control group, indicating that the resting EEG power spectrum of low-risk LPIs did not indicate abnormal manifestations.

Various brain cognitive abilities must be achieved through the combined effect of multiple brain regions. The connectivity study in the two LPI groups suggests that the functional connectivity between related brain regions was lower than that of full-term infants, which is also one of the reasons for the lack of brain cognitive function in the early stage after LPIs birth. Meijer proposed that changes in coherence values could reflect the functional status of specific neuronal connections (such as corpus callosum connections, thalamocortical connections, and other interneuronal networks) [39]. Duffy found that healthy infants born near term had greater connectivity between frontal and occipital regions and left central and temporal regions compared with preterm infants [40]. Grieve analyzed the coherence of functional connectivity and found that, compared with full-term infants, the interhemispheric coherence of the frontal polar region and parietal occipital region in the 1–12 Hz frequency band was significantly reduced in very low birth weight infants [41]. Our study on LPIs found a similar performance in the 1–8 Hz frequency band.

GSD showed that there was no significant difference in gross motor development in high-risk LPIs at the age of 1 year after correction, but fine motor function, language, personal social interaction and adaptability were all delayed. There was abnormal brain cognitive development in high-risk LPIs at the age of 1 year, which should receive close attention. The earlier the intervention time, the better the effect may be. There is a need to capture abnormal signals in early birth; thus, we explored the early predictors of brain cognitive function development in LPIs. As mentioned above, ERP can better reflect the lag of brain cognitive function in LPIs in the early stage; therefore, we examined the correlation between MMR and the Gesell test. According to the sensitivity and specificity of MMR amplitude in predicting prognosis and AUC results, MMR amplitude can be used as an effective indicator for early prediction of brain cognitive development prognosis at the age of 1 year.

This study has shortcomings in assessing brain cognitive function in the early stage of LPIs. Firstly, the coherence of the functional connectivity of resting EEG is sensitive to the volume conduction effect, and the closer the distance between the two electrodes, the greater the effect, which makes the connectivity of the cortical network at the level of the scalp and source space blurred. In addition, neonates, especially premature infants, have a smaller head circumference and the distance between the 19 channels may be smaller. Therefore, we cannot completely exclude the influence of the volume conduction effect in this study. Secondly, the quiet and awake state of newborns is relatively short, and recording a little EEG during sleep is inevitable. However, studies have demonstrated that both active sleep and quiet sleep have an impact on resting EEG and connectivity [42], this study did not assess the influence of this factor in depth. Thirdly, perinatal diseases and high-risk factors such as asphyxia, infection and hypoglycemia have different mechanisms in brain cognitive impairment, which will be explored one by one in the next phase of this study. Fourthly, functional magnetic resonance imaging (fMRI) technology is gradually maturing, and numerous research results have been achieved in the field of cognitive function. The combination of neuroelectrophysiological technology and fMRI in the exploration of brain function is sure to produce more important findings. Subsequent investigations ought to concentrate on formulating tailored intervention approaches, implementing long-term developmental monitoring, incorporating sophisticated neuroimaging methodologies, examining risk and protective elements, and accentuating prompt detection and intervention in order to enhance cognitive performance and social adaptability in LPIs.

## Conclusion

Our findings revealed that the cognitive function of LPI lags behind that of full-term infants in early life. Preterm birth and perinatal diseases or high risk factors affected brain cognitive function in LPIs. MMR amplitude can be used as an early predictor of brain cognitive development in LPIs.

## Data Availability

The full data set and other materials related to about this study can be obtained from the corresponding author on reasonable request.

## Abbreviations

LPIs: Late preterm infants
ERP: Event-related potential
EEG: Electroencephalography
RS-EEG: Resting electroencephalography
GDS: Gesell Developmental Scale
MMR: Mismatch response
BSID: Bayley Scales of Infant Development
SPL: Sound Pressure Level
fMRI: Functional magnetic resonance imaging

## References

[1] Zhanghua Y, Jihong Q, Bei W, Tianwen Z, Dongying Z, Yonghong Z (2015). Prospective study of short-tem Complications and intellectual development in late pretem infants. Chin J Neonatology.

[2] Ballantyne M, Benzies KM, McDonald S, Magill-Evans J, Tough S (2016). Risk of developmental delay: comparison of late preterm and full term Canadian infants at age 12 months. Early Hum Dev.

[3] Cheong JL, Doyle LW, Burnett AC, Lee KJ, Walsh JM, Potter CR, et al. Association between Moderate and late Preterm Birth and Neurodevelopment and Social-Emotional Development at Age 2 years. Jama Pediatr. 2017;171(4). 10.1001/jamapediatrics.2016.4805. 10.1001/jamapediatrics.2016.4805 28152144

[4] Woythaler M, McCormick MC, Mao WY, Smith VC (2015). Late Preterm infants and neurodevelopmental outcomes at Kindergarten. Pediatrics.

[5] Allan NP, Hume LE, Allan DM, Farrington AL, Lonigan CJ (2014). Relations between inhibitory control and the development of academic skills in preschool and kindergarten: a meta-analysis. Dev Psychol.

[6] Ferronato PA, Domellof E, Ronnqvist L (2014). Early influence of auditory stimuli on upper-limb movements in young human infants: an overview. Front Psychol.

[7] Novitski N, Huotilainen M, Tervaniemi M, Naatanen R, Fellman V (2007). Neonatal frequency discrimination in 250-4000-Hz range: electrophysiological evidence. Clin Neurophysiol.

[8] Näätänen R, Alho K (1997). Mismatch negativity—the measure for central sound representation accuracy. Audiol Neuro-Otology.

[9] He C, Hotson L, Trainor LJ (2017). Mismatch responses to pitch changes in early infancy. J Cogn Neurosci.

[10] Morr ML, Shafer VL, Kreuzer JA (2002). Maturation of Mismatch Negativity in typically developing infants and Preschool Children. Ear & Hearing.

[11] Lovio R, Naatanen R, Kujala T (2010). Abnormal pattern of cortical speech feature discrimination in 6-year-old children at risk for dyslexia. Brain Res.

[12] Huttunen-Scott T, Kaartinen J, Tolvanen A, Lyytinen H (2008). Mismatch negativity (MMN) elicited by duration deviations in children with reading disorder, attention deficit or both. Int J Psychophysiol.

[13] Tanaka M, Okubo O, Fuchigami T, Harada K (2001). A study of mismatch negativity in newborns. Pediatr Int.

[14] Bisiacchi PS, Mento G, Suppiej A (2009). Cortical auditory processing in preterm newborns: an ERP study. Biol Psychol.

[15] Ragó A, Honbolygó F, Róna Z, Beke A, Csépe V (2013). Effect of maturation on suprasegmental speech processing in full- and preterm infants: a mismatch negativity study. Res Dev Disabil.

[16] Cheng YY, Lee CY. The development of mismatch responses to Mandarin Lexical Tone in 12-to 24-Month-Old infants. Front Psychol. 2018;9. 10.3389/fpsyg.2018.00448. 10.3389/fpsyg.2018.00448 PMC5902780 29692746

[17] Kushnerenko EV, Van dBBRH (2013). István. W. separating acoustic deviance from novelty during the first year of life: a review of event-related potential evidence. Front Psychol.

[18] Loo SK, Lenartowicz A, Makeig S, Research Review (2016). Use of EEG biomarkers in child psychiatry research - current state and future directions. J Child Psychol Psychiatry.

[19] Almubarak S, Wong PKH (2011). Long-term clinical outcome of neonatal EEG findings. J Clin Neurophysiol.

[20] Périvier M, Rozé J, Gascoin G, Hanf M, Branger B, Rouger V (2015). Neonatal EEG and neurodevelopmental outcome in preterm infants born before 32weeks. Archives of Disease in Childhood - Fetal Neonatal Ed.

[21] Roth JK, Johnson MK, Tokoglu F, Murphy I, Constable RT. Modulating intrinsic connectivity: adjacent subregions within supplementary motor cortex, Dorsolateral Prefrontal Cortex, and parietal cortex connect to separate functional networks during Task and also connect during Rest. PLoS ONE. 2014;9(3). 10.1371/journal.pone.0090672. 10.1371/journal.pone.0090672 PMC3956459 24637793

[22] Anderson AJ, Perone S (2018). Developmental change in the resting state electroencephalogram: insights into cognition and the brain. Brain Cogn.

[23] Brito NH, Fifera WP, Myers MM, Elliott AJ, Noble KG (2016). Associations among family socioeconomic status, EEG power at birth, and cognitive skills during infancy. Dev Cogn Neurosci.

[24] Williams IA, Tarullo AR, Grieve PG, Wilpers A, Vignola EF, Myers MM (2012). Fetal cerebrovascular resistance and neonatal EEG predict 18-month neurodevelopmental outcome in infants with congenital Heart Disease. Ultrasound in Obstetrics & Gynecology.

[25] Benasich AA, Gou Z, Choudhury N, Harris KD (2008). Early cognitive and language skills are linked to resting frontal gamma power across the first 3 years. Behav Brain Res.

[26] Marlow N, Wolke D, Bracewell MA, Samara M (2005). Neurologic and developmental disability at six years of age after extremely preterm birth. N Engl J Med.

[27] Pavlidis E, Lloyd RO, Boylan GB (2017). EEG – a valuable biomarker of brain injury in preterm infants. Dev Neurosci.

[28] Doesburg SM, Ribary U, Herdman AT (2016). Altered long-range alpha-band synchronization during visual short-term memory retention in children born very preterm. NeuroImage: Clin.

[29] Bisiacchi PS, Mento G, Suppiej AJBP. Cortical auditory processing in preterm newborns: an ERP study. 2009;82(2):176–85. 10.1016/j.biopsycho.2009.07.005 19631252

[30] Leipala JA, Partanen E, Kushnerenko E, Huotilainen M, Fellman V (2011). Perinatal cerebral insults alter auditory event-related potentials. Early Hum Dev.

[31] deReginer RA, Nelson CA, Thomas KM, Wewerka S, Georgieff MK (2000). Neurophysiologic evaluation of auditory recognition memory in healthy newborn infants and infants of diabetic mothers. J Pediatr.

[32] Anderson AJ, Perone SJB, editors. cognition. Developmental change in the resting state electroencephalogram: Insights into cognition and the brain. 2018;126(OCT.):40–52. 10.1016/j.bandc.2018.08.001 30144749

[33] Noreika V, Georgieva S, Wass S, Leong V. 14 challenges and their solutions for conducting social neuroscience and longitudinal EEG research with infants. Infant Behav Dev. 2020;58. 10.1016/j.infbeh.2019.101393. 10.1016/j.infbeh.2019.101393 31830682

[34] Okumura A, Kubota T, Toyota N, Kidokoro H, Maruyama K, Kato T (2003). Amplitude spectral analysis of maturational changes of delta waves in preterm infants. Brain Dev.

[35] Bell AH, McClure BG, McCullagh PJ, McClelland RJ (1991). Variation in power spectral analysis of the EEG with gestational age. J Clin Neurophysiol.

[36] Tsuchida TN, Wusthoff CJ, Shellhaas RA, Abend NS, Hahn CD, Sullivan JE (2013). American Clinical Neurophysiology Society standardized EEG terminology and categorization for the description of continuous EEG monitoring in neonates: report of the American Clinical Neurophysiology Society Critical Care Monitoring Committee. J Clin Neurophysiol.

[37] Begus K, Bonawitz E. The rhythm of learning: Theta oscillations as an index of active learning in infancy. Dev Cogn Neurosci. 2020;45. 10.1016/j.dcn.2020.100810. 10.1016/j.dcn.2020.100810 PMC7371744 33040970

[38] Niemarkt HJ, Jennekens W, Pasman JW, Katgert T, van Pul C, Gavtlanes AWD, 32 (2011). Maturational changes in Automated EEG Spectral Power Analysis in Preterm infants. Pediatr Res.

[39] Meijer EJ, Hermans KHM, Zwanenburg A, Jennekens W, Niemarkt HJ, Cluitmans PJM (2014). Functional connectivity in preterm infants derived from EEG coherence analysis. Eur J Pediatr Neurol.

[40] Duffy FH, Als H, McAnulty GB (2003). Infant EEG spectral coherence data during quiet sleep: unrestricted principal components analysis - relation of factors to gestational age, medical risk, and neurobehavioral status. Clin Electroencephalogr.

[41] Grieve PG, Isler JR, Izraelit A, Peterson BS, Fifer WP, Myers MM (2008). EEG functional connectivity in term age extremely low birth weight infants. Clin Neurophysiol.

[42] Gonzalez JJ, Manas S, De Vera L, Mendez LD, Lopez S, Garrido JM (2011). Assessment of electroencephalographic functional connectivity in term and preterm neonates. Clin Neurophysiol.

